# Facilitating motor imagery-based brain–computer interface for stroke patients using passive movement

**DOI:** 10.1007/s00521-016-2234-7

**Published:** 2016-03-04

**Authors:** Mahnaz Arvaneh, Cuntai Guan, Kai Keng Ang, Tomas E. Ward, Karen S. G. Chua, Christopher Wee Keong Kuah, Gopal Joseph Ephraim Joseph, Kok Soon Phua, Chuanchu Wang

**Affiliations:** 1 Department of Automatic Control and Systems Engineering, University of Sheffield, Sheffield, UK; 2 Institute for Infocomm Research, Agency for Science, Technology and Research (A*STAR), Singapore, Singapore; 3 Department of Electronic Engineering, National University of Ireland, Maynooth, Ireland; 4 Department of Rehabilitation Medicine, Tan Tock Seng Hospital, Singapore, Singapore

**Keywords:** Adaptation, Brain–computer interface, Motor imagery, Passive movement, Stroke rehabilitation

## Abstract

Motor imagery-based brain–computer interface (MI-BCI) has been proposed as a rehabilitation tool to facilitate motor recovery in stroke. However, the calibration of a BCI system is a time-consuming and fatiguing process for stroke patients, which leaves reduced time for actual therapeutic interaction. Studies have shown that passive movement (PM) (i.e., the execution of a movement by an external agency without any voluntary motions) and motor imagery (MI) (i.e., the mental rehearsal of a movement without any activation of the muscles) induce similar EEG patterns over the motor cortex. Since performing PM is less fatiguing for the patients, this paper investigates the effectiveness of calibrating MI-BCIs from PM for stroke subjects in terms of classification accuracy. For this purpose, a new adaptive algorithm called filter bank data space adaptation (FB-DSA) is proposed. The FB-DSA algorithm linearly transforms the band-pass-filtered MI data such that the distribution difference between the MI and PM data is minimized. The effectiveness of the proposed algorithm is evaluated by an offline study on data collected from 16 healthy subjects and 6 stroke patients. The results show that the proposed FB-DSA algorithm significantly improved the classification accuracies of the PM and MI calibrated models (*p* < 0.05). According to the obtained classification accuracies, the PM calibrated models that were adapted using the proposed FB-DSA algorithm outperformed the MI calibrated models by an average of 2.3 and 4.5 % for the healthy and stroke subjects respectively. In addition, our results suggest that the disparity between MI and PM could be stronger in the stroke patients compared to the healthy subjects, and there would be thus an increased need to use the proposed FB-DSA algorithm in BCI-based stroke rehabilitation calibrated from PM.

## Introduction

A brain–computer interface (BCI) provides a direct communication pathway between a human brain and an external device [1, 2]. Using appropriate sensors and data processing algorithms, a BCI maps patterns of brain activity associated with a volitional thought onto signals suitable for communication and control [3, 4]. Such technology holds great promise as a basis for assisting people with severe communication and motor disabilities. More recently, BCI systems have been adapted to operate so as to encourage neurophysiological activity that might promote motor recovery in conditions such as stroke [5–7]. Several studies have demonstrated that motor imagery (MI) has a positive effect on motor rehabilitation after stroke through activation of the affected sensorimotor networks [8–10]. Since the performance of MI is internal to the subject, and thus not directly observable, BCI can facilitate the MI-based stroke rehabilitation by providing direct and immediate feedback on the MI performance.

In most of BCI systems, brain signals are measured by electroencephalogram (EEG), due to its low cost and high temporal resolution [2]. However, the EEG patterns used for discerning MI vary considerably between sessions even for the same subject [11]. Thus, MI-BCIs typically require the recording of labeled training data (acquired without giving feedback to the patient) during a so called calibration phase at the beginning of each session. The calibration phase takes around 20–30 min, and is thus time-consuming and tedious, particularly for patients who require long-term BCI therapy. Hence, algorithms that require less calibration time is highly desirable for patients use.

Several approaches have been proposed in the literature to remove the calibration phase, for example, through concatenating and clustering historic spatial filters and data from the same subject [12–14], or by creating an ensemble of historic spatial filters and classifiers derived from different subjects [15]. These methods, however, require a large amount of historic data to be available. Other techniques have sought to reduce the calibration phase through the use of co-adaptive methods or semi-supervised learning approaches [16–21]. These methods may initially have a limited performance, but it improves after a considerable adaptation time. Despite several studies in this issue, reducing the calibration time has still remained a challenging issue.

Previous research studies have demonstrated that specific EEG synchronization (ERS), are largely similar during passive movement (PM) and MI [22–24]. Therefore, PM could potentially serve as a repeatable, and observable input to produce the stereotypical EEG patterns required for calibrating a BCI model. Since PM exercises are a part of normal stroke rehabilitation [25], MI-based BCI rehabilitation therapy could start immediately by training the classifier using the PM data collected in the previous physical therapy session. The issue is that PM-induced EEG patterns may not be identical to those produced during MI [24, 26], and further, due to other inter-session variations (e.g., electrode positioning, cognitive state etc), the use of adaptive methods may be required to enhance the performance of a BCI system calibrated in this way.

To address this issue, this paper proposes a new filter bank data space adaptation (FB-DSA) algorithm to linearly transform the filter bank band-passed MI data, such that the distribution difference between the PM and MI data is minimized. This algorithm is a modified version of our previously proposed algorithm called EEG data space adaptation [27]. The performance of the proposed FB-DSA algorithm is evaluated on data collected from 6 stroke and 16 healthy subjects. The experiments performed for both the stroke and healthy subjects are based on those previously practised for MI-BCI in stroke rehabilitation [5]. For the first time, this paper also provides evidence supporting that the disparity between PM and MI is significantly stronger in the stroke patients compared to the healthy subjects, and it would thus increase the need to use adaptive algorithms such as the proposed FB-DSA algorithm in BCI-based stroke rehabilitation calibrated from PM data.

## Methodology

### Filter bank common spatial patterns (FBCSP)

Recently, the FBCSP algorithm [28] was proposed that combined a filter bank framework with the common spatial patterns (CSP) algorithm [29] to select the most discriminative features using a mutual information-based criterion [30]. In this paper FBCSP was used to classify the EEG data as it was the basis of all the winning algorithms in the EEG category of the BCI competition IV [31]. The FBCSP algorithm comprises the following steps:

1. Spectral filtering: This step uses a filter bank that decomposes the EEG data using nine equal bandwidth filters, namely 4–8, 8–12, …, 36–40 Hz. These frequency ranges cover most of the manually or heuristically selected settings used in the literature.
2. Spatial filtering: In this step, the EEG data from each frequency band are spatially filtered using the CSP algorithm. Let ${\mathbf{x }}_{\rm b}\in {\mathbf{R }}^{n \times s}$ represent a single-trial EEG data from the *b*th band-pass filter, where *n* and *s* denote the number of channels and the number of measurement samples respectively. The CSP matrix linearly transforms ${\mathbf{x}}_{\rm b}$ to spatially filtered ${\mathbf{Z}} _{\rm b}$ as
  1 $$\begin{aligned} {\mathbf {Z}}_{\rm b}={\mathbf{W }}_{\rm b}\;{\mathbf{x }}_{\rm b}, \end{aligned}$$
  where ${\mathbf {W}}_{\rm b}\in {\mathbb {R}}^{n\times n}$ denotes the CSP matrix. ${\mathbf {W}}_{\rm b}$, is generally computed by solving the eigenvalue decomposition problem:
  2 $$\begin{aligned} {\mathbf {C}}_{{\rm b},1}{\mathbf {W}}_{\rm b} = \left( {\mathbf {C}}_{{\rm b},1} + {\mathbf {C}}_{{\rm b},2} \right) {\mathbf {W}}_{\rm b}{\mathbf {D}}, \end{aligned}$$
  where ${\mathbf {C}}_{{\rm b},1}$ and ${\mathbf {C}}_{{\rm b},2}$ are respectively the averaged covariance matrices of the band-passed EEG data of each class; ${\mathbf {D}}$ is the diagonal matrix that contains the eigenvalues of $({\mathbf {C}}_{{\rm b},1}+{\mathbf {C}}_{{\rm b},2})^{-1}{\mathbf {C}}_{{\rm b},1}$. A direction that has a large variance in events of one class (high eigenvalue) has a small variance in events of the other class (low eigenvalue). Usually, only the first and last *m* rows of ${\mathbf {W}}_{\rm b}$, corresponding to the highest and lowest eigenvalues, are used as the most discriminative filters to perform spatial filtering [32].
3. Feature extraction: The *m* pairs of the CSP features corresponding to the *i*th trial from the *b*th band-pass filter are computed as [32]
  3 $$\begin{aligned} {\mathbf {f}}_{{\rm b},i}={{\rm log}}\left( {{\rm diag}}\left( {\mathbf {{z}}}_{{\rm b},i} {\mathbf {{z}}}^{{{\rm T}}}_{{\rm b},i}\right) /{{\rm tr}}\left[ {\mathbf {{z}}}_{{\rm b},i} {\mathbf {{z}}}^{{{\rm T}}}_{{\rm b},i}\right] \right) , \end{aligned}$$
  where ${\mathbf {f}}_{{\rm b},i}\in {\mathbb {R}}^{1\times 2m}$; ${\mathbf {{z}}}_{{\rm b},i}$ represents the first and the last *m* rows of ${\mathbf {Z}}_{\rm b}$; diag(.) returns the diagonal elements of the square matrix; tr[.] returns the sum of the diagonal elements of the square matrix; and the superscript T denotes the transpose operator. Since the nine frequency bands are used, the feature vector for the *i*th trial is formed as
  4 $$\begin{aligned} {\mathbf {F}}_{i}=[{\mathbf {f}}_{1,i}\;,{\mathbf {f}}_{2,i}\;,\ldots \;, {\mathbf {f}}_{9,i}], \end{aligned}$$
  where ${\mathbf {F}}_{i}\in {\mathbb {R}}^{1\times 18m}$. In this study, $m=2$ pairs of the spatial filters were used as suggested in [28].
4. Feature selection: The last step selects four pairs of the features from the feature vector **F** as the most discriminative features using the mutual information-based best individual feature (MIBIF) algorithm [30]. The selected features are used as the inputs to the classifier.

### Filter bank data space adaptation (FB-DSA)

In this work, the set of the labeled EEG trials in the calibration session filtered by the *b*th band-pass filter is denoted as $\bar{D_{\rm b}}=\{({\bar{\mathbf{x}}}_{{\rm b},i},{\bar{\mathbf{y }}}_{i})\}_{i=1}^{\bar{N}}$, where ${\bar{{\mathbf{x }}}}_{{\rm b},i}\in {\bar{{\mathbf{X }}}}_{\rm b}\subset {\mathbb {R}}^{n \times s}$ denotes the *i*th single-trial EEG filtered by the *b*th filter, and ${\bar{\mathbf{y }}}_{i}\in {\bar{\mathbf{Y }}}\subset {\mathbb {R}}$ is the class label of the *i*th single-trial EEG. In the evaluation session, the available labeled EEG trials from the *b*th band-pass filter are denoted as $D_{\rm b}=\{({\mathbf{x }}_{{\rm b},i},{\mathbf{y }}_{i})\}_{i=1}^{N}$, where ${\mathbf{x }}_{{\rm b},i}\in {\mathbf{X }}_{\rm b}\subset {\mathbb {R}}^{n \times s}$, and ${\mathbf{y }}_{i}\in \mathbf{Y }\subset {\mathbb {R}}$.

The dissimilarities between the calibration and evaluation sessions from the *b*th band-pass filter can yield different joint distributions for the corresponding evaluation session $P({\mathbf{X }}_{\rm b},{\mathbf{Y }})$ and calibration session $P({\bar{\mathbf{X}}}_{\rm b},{\bar{\mathbf{Y}}})$. However, changing the representation of ${\mathbf{X }}_{\rm b}$, while the representation of **Y** is fixed, can change the joint distribution of the evaluation session. Following this concept, assume $g:{\mathbf{X }}_{\rm b}\longrightarrow {\mathbf{H }}_{\rm b}$ as a function that transforms a band-pass-filtered single-trial EEG, ${\mathbf{x }}_{\rm b}$, from the evaluation space into another space ${\mathbf{h }}_{\rm b}=g({\mathbf{x }}_{\rm b})\in {\mathbf{H}}_{\rm b}$. Thus, if for each band-pass filter, a transformation function *g* can be computed to yield the same joint distributions for both the calibration and evaluation sessions $P({\mathbf{H}}_{\rm b},{\mathbf{Y}})=P({\bar{{\mathbf{X }}}}_{\rm b},\bar{\mathbf{Y }})$, the optimal model that classifies the calibration session will be still optimal for classifying the evaluation session.

For this purpose, a linear transformation function is proposed as

5 $$\begin{aligned} {\mathbf{h}}_{\rm b}={\mathbf{V}}_{\rm b}^{{{\rm T}}}{\mathbf{x }}_{\rm b}, \end{aligned}$$

where ${\mathbf{V}}_{\rm b}\in {\mathbb {R}}^{n \times n}$ denotes the FB-DSA transformation matrix. The transformation matrix ${\mathbf{V}}_{\rm b}$ should be computed such that the distribution difference between the evaluation session and the calibration session filtered by the *b*th band-pass filter is reduced.

Similar to [33], we assume that the differences between the calibration and evaluation sessions can be observed in the first two moments of the single-trial EEG (i.e., mean and covariance). Following this assumption, to simplify the problem, we only compare the average distributions of the EEG trials between the calibration session and the evaluation session to compute a transformation matrix that minimizes the differences between their first two moments.

Since the single-trial EEG is band-pass-filtered, it has approximately zero mean value. Consequently, the average distribution of a group of band-pass-filtered EEG trials can be defined by a zero mean and a covariance matrix computed from averaging the covariance matrices over the multiple EEG trials. Based on the maximum entropy principle, the most prudent model for modeling the distribution of the single-trial EEG that is consistent with zero mean and a covariance matrix is Gaussian [33]. Thus, the Kullback–Leibler (KL) divergence between gaussians can be used to measure the difference between the distributions.

The KL divergence between the distributions of two groups of band-pass-filtered EEG trials, presented as $N_{0}(0,{{\varvec{\Sigma }}})$ and $N_{1}(0,\overline{{{\varvec{\Sigma }}}})$ (taken as reference), has a closed form expression

6 $$\begin{aligned} {{\rm KL}}[N_{0}||N_{1}]=\frac{1}{2}\left[ {{\rm tr}} (\overline{{{\varvec{\Sigma }}}}^{-1}{{\varvec{\Sigma }}}) -\ln \left( \frac{\det ({{\varvec{\Sigma }}})}{\det (\overline{{{\varvec{\Sigma }}}})}\right) -d\right] , \end{aligned}$$

where $\overline{{{\varvec{\Sigma }}}}$ and ${{\varvec{\Sigma }}}$ denote the average covariance matrices of the two groups of the EEG trials; $\det$ and *d* denote the determinant function and the dimensionality of the data respectively.

Let $N(0,\overline{{{\varvec{\Sigma }}}}_{{\rm b},j})$ be the average distribution of the *b*th band-pass-filtered EEG trials belonging to the class *j* in the calibration session. Using the available labeled trials from the evaluation session $D_{\rm b}=\{({\mathbf{x }}_{{\rm b},i},{\mathbf{y}}_{i})\}_{i=1}^{N}$, the average distribution of the transformed EEG trials belonging to the class *j* and the *b*th filter is estimated as $N(0,{\mathbf{V}}_{\rm b}^{{{\rm T}}}{{\varvec{\Sigma }}}_{{\rm b},j} {\mathbf{V}}_{\rm b})$, where ${\mathbf{V}}_{\rm b}$ denotes the linear transformation matrix for the *b*th filtered data, and ${{\varvec{\Sigma }}}_{{\rm b},j}$ denotes the average covariance matrix of class *j* in the evaluation session estimated using $D_{\rm b}$. When the class probabilities are balanced, using the KL divergence the optimal ${\mathbf{V}}_{\rm b}$ can be computed as the solution of the minimization problem

7 $$\begin{aligned} \underset{{\mathbf{V}}_{\rm b}}{\text {min}}\;\;{{\rm L}}_{\rm b}({\mathbf{V}}_{\rm b})&=\underset{{\mathbf{V}}_{\rm b}}{\text {min}}\sum _{j=1}^{2}{\rm KL}\left[ N(0,{\mathbf{V}}_{\rm b}^{{{\rm T}}}{{\varvec{\Sigma }}}_{{\rm b},j}{\mathbf{V}}_{\rm b})||N(0,\overline{{{\varvec{\Sigma }}}}_{{\rm b},j})\right] \\ &=\underset{{\mathbf{V}}_{\rm b}}{\text {min}} \sum _{j=1}^{2}\frac{1}{2}\left[ {\rm tr}({\overline{{{\varvec{\Sigma }}}}_{{\rm b},j}}^{-1}{{\mathbf{V }}}_{\rm b}^{{{\rm T}}}{{\varvec{\Sigma }}}_{{\rm b},j}{{\mathbf{V }}}_{\rm b})\right. \\ &\quad-\,\left.\ln \left( \frac{\det ({{\mathbf{V }}}_{\rm b}^{{{\rm T}}} {{{\varvec{\Sigma }}}}_{{\rm b},j}{{\mathbf{V }}}_{\rm b})}{\det (\overline{{{\varvec{\Sigma }}}}_{{\rm b},j})}\right) -d\right].\\ \end{aligned}$$

To minimize (7), it is sufficient to calculate the first order derivative of the loss function ${{\rm L}}_{\rm b}({{\mathbf{V }}}_{\rm b})$ with respect to ${{\mathbf{V }}}_{\rm b}$, and set it to zero;

8 $$\begin{aligned} \frac{{\rm d}{{\rm L}}_{\rm b}}{{\rm d}{{\mathbf{V }}}_{\rm b}}=\sum _{j=1}^{2}\frac{1}{2}\frac{\rm d}{{\rm d}{{\mathbf{V }}}_{\rm b}}\left[ {\rm tr}\left( {\overline{{{\varvec{\Sigma }}}}_{{\rm b},j}}^{-1}{{\mathbf{V }}}_{\rm b}^{{{\rm T}}}{{{\varvec{\Sigma }}}}_{{\rm b},j}{{\mathbf{V }}}_{\rm b}\right) -\ln \left( \det \left( {{\mathbf{V }}}_{\rm b}^{{{\rm T}}}{{{\varvec{\Sigma }}}}_{{\rm b},j}{{\mathbf{V }}}_{\rm b}\right) \right) \right] . \end{aligned}$$

Thus, one solution for (8) is when (see [27] for more details)

9 $$\begin{aligned} {\mathbf{V }}^{*}_{\rm b}=\sqrt{2}\left( \left( {\overline{{{\varvec{\Sigma }}}}_{{\rm b},1}}^{-1}{{{\varvec{\Sigma }}}}_{{\rm b},1}+{\overline{{{\varvec{\Sigma }}}}_{{\rm b},2}}^{-1}{{{\varvec{\Sigma }}}}_{{\rm b},2}\right) ^{\dag }\right) ^{0.5}, \end{aligned}$$

where $\dag$ denotes the pseudoinverse of the matrix. ${\mathbf{V }}^{*}_{\rm b}$ is the optimal linear transformation matrix computed for the *b*th filter in the FB-DSA algorithm. Therefore, ${\mathbf{V}}^{*}_{\rm b}$ linearly transforms the EEG data of the *b*th filter from the evaluation session to the corresponding calibration session, such that the distribution difference between these sessions is minimized. As expected, in the case that the calibration and the evaluation data have similar distributions, (i.e., the average covariance matrices of the corresponding classes are equal), ${\mathbf{V}}^{*}_{\rm b}$ is the identity matrix.

**Fig. 1:**
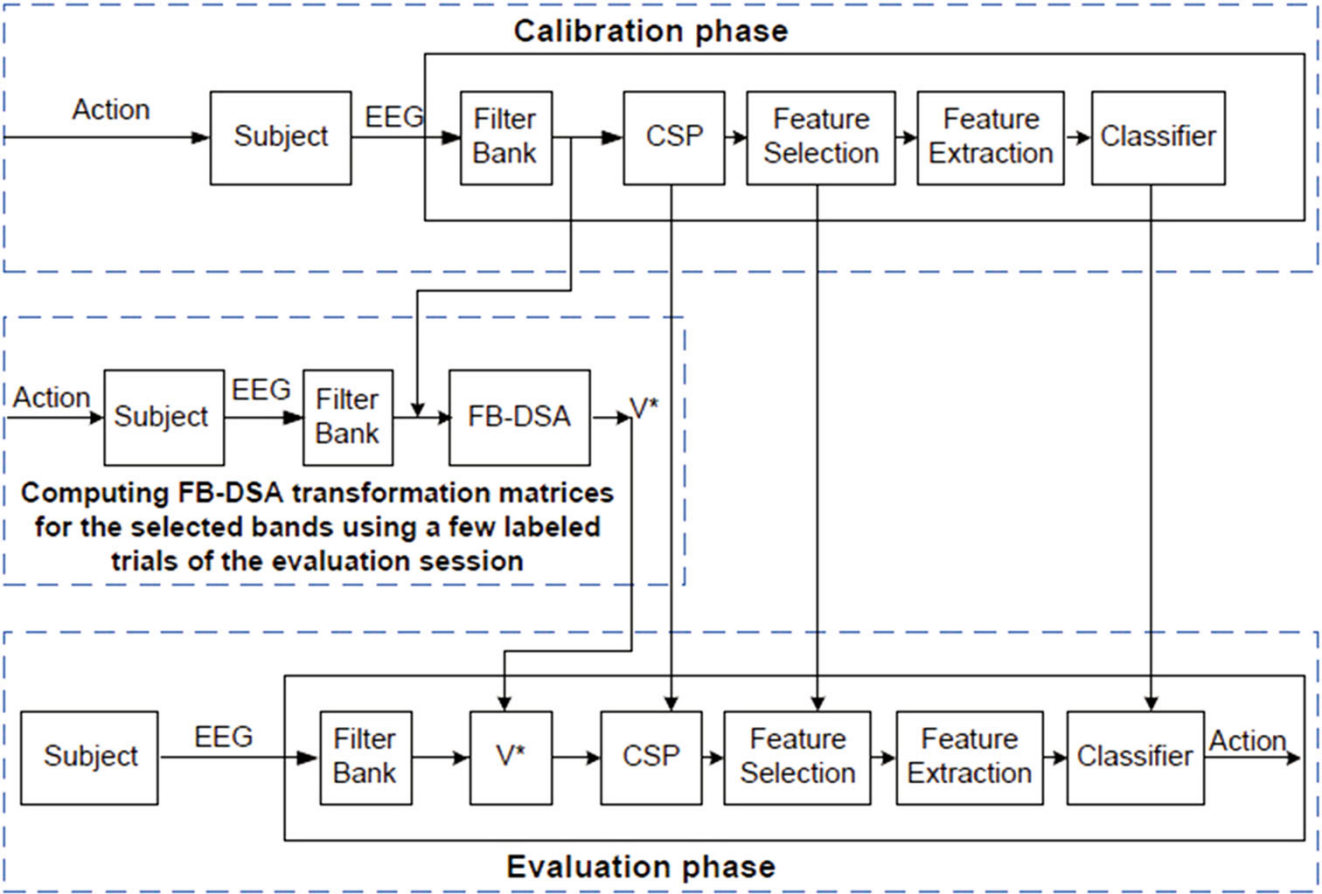
Architecture of the FB-DSA algorithm in the FBCSP framework for the calibration and evaluation phases

The architecture of the proposed FB-DSA algorithm in the FBCSP framework is demonstrated in Fig. 1. In the calibration phase, the FBCSP algorithm is used to train a subject-specific model using PM or MI data. In the evaluation phase, the new trials from each band-pass filter are optimally transformed by their corresponding FB-DSA transformation matrix computed using a few past EEG trials of the evaluation session (i.e., 20 trials in this study). Subsequently, the transformed FB-DSA trials are directly applied to the corresponding CSP filters and the classifier trained in the calibration phase. To avoid irrelevant computation, it suffices to compute the FB-DSA transformation matrices only for the frequency bands which have features selected for the classification.

## Experiments

### Subjects

This study recruited 18 healthy subjects and seven hemiparetic stroke patients. Ethics committee approval was obtained from the institution’s Domain Specific Review Board, National Healthcare Group, Singapore. Informed consent were obtained from all the participants prior the study enrollment. One of the stroke patients could not commit to the whole study. Thus, the corresponding data were excluded. The experiments performed for both the stroke and healthy subjects were based on those previously practised for EEG-based MI-BCI in stroke rehabilitation [5]. From among the healthy subjects, two subjects chose to perform MI and PM of the left hand while the remaining 16 subjects chose to perform on the right hand. For four patients, the stroke affected their left hand, while the right hand was affected in the remaining two patients.

### Data description

#### Dataset collected from the healthy subjects

**Fig. 2:**
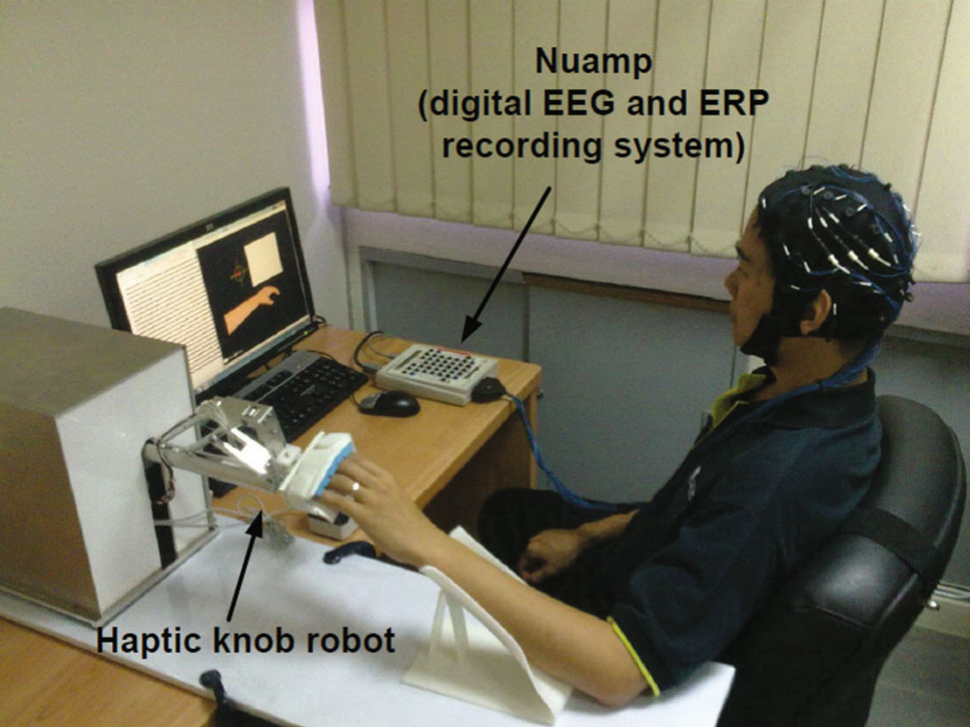
Experimental setup to collect EEG data from passive movement of the left hand using the haptic knob robot [34] for calibrating the EEG-based motor imagery BCI

EEG from 27 channels was collected. The subjects were instructed to minimize physical movements and eye blinking throughout the EEG recording process. For each subject, EEG data were collected without feedback in two measurement sessions conducted on separate days. For the first day, four runs of EEG data were collected. In the first two runs, the subjects were instructed to perform MI of the chosen hand and background rest condition. Subsequently, in the next two runs, the subjects engaged in PM of the chosen hand using the haptic knob robot [34], and background rest condition. Figure 2 shows the experimental setup to collect EEG data, as the haptic knob robot is used to move the subject’s left hand.

**Fig. 3:**
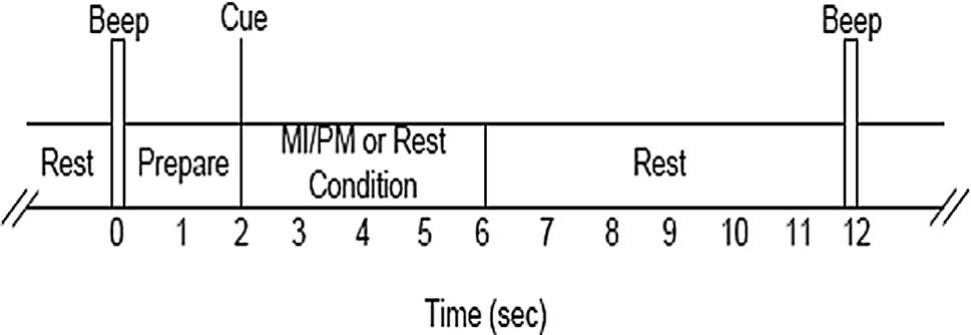
Timing of each trial including performing MI/PM of the hand or background rest tasks

The subjects were instructed to perform kinesthetic MI of the chosen hand during the first two runs. The subjects were also instructed to perform mental counting during the background rest condition. In the last two runs, the subjects were asked to relax while the movement of the chosen hand was performed using the haptic knob robot [34]. The instructions were on the computer screen in each trial. As shown in Fig. 3, each trial lasted for 12 s, as the subject was first prepared with a cue for 2 s, then an “action” command instructed the subject for 4 s, and finally the subject was asked to rest for 6 s. Each run comprised of 40 trials of either MI or PM, and 40 trials of background rest condition. Considering the EEG set up time, the practice time, and the rest time between the blocks, the session on the first day took around an hour and 45 min. The EEG data from the first and second runs were used to calibrate a subject-specific model referred to as the MI model, and subsequently the EEG data from the third and fourth runs were used to calibrate a subject-specific model referred to as the PM model.

In the second day of this study, three runs of the EEG data were collected without feedback from each subject while performing MI of the chosen hand and background rest condition. Each run again comprised of 40 trials of MI and 40 trials of background rest condition. The session on the second day took around an hour and a half in total. The EEG data collected from these three runs were used to evaluate the calibrated models from the first day.

Before calibrating the subject-specific models, $10\times 10$-fold cross-validation accuracies of the first two runs as well as the last two runs recorded on the first day were calculated to find those subjects performing either MI or PM at chance level. Using the inverse of the binomial cumulative distribution function with 95 % confidence, the accuracy on the respective action at chance level is approximately 43–57 %. Hence, those subjects whose PM or MI data have $10\times 10$-fold cross-validation accuracies between 43 and 57 % can be excluded as their data are not proper enough for calibrating a model. The results showed that two subjects from the 18 aforementioned subjects performed MI and PM at chance level. Hence, these two subjects were removed, and the remaining 16 subjects were used for this study.

#### Dataset collected from the stroke patients

Table 1 provides more clinical information about the six stroke patients. The protocol used for EEG recording from the stroke subjects was very similar to that used for the healthy subjects. The minor differences are as follows: During the experiments, the patients performed either MI or PM of the stroke affected hand rather than the chosen hand. In addition, in the second day of this study, two runs of the EEG data were recorded from each subject while performing MI of the affected hand and rest condition without feedback. Similar to the healthy subjects, the EEG data recorded on the second day were used to evaluate the subject-specific MI and PM models calibrated using the data collected on the first day.

**Table 1:** Demographic and clinical information for *N* = 6 stroke subjects who participated in this study

Gender	Type	Stroke			Duration since	FMA
		Side	Nature	Mean		
M/F	I/H	R/L	C/S	Age	Stroke (days)	(Week 0)
4M	2I	2R	1C	54.0 $\pm$ 8.9	285.7 $\pm$ 64	33.0 $\pm$ 16.2

*M* male, *F* female, *I* infarction, *H* hemorrhagic, *R* right, *L* left, *C* cortical, *S* subcortical, *FMA* Fugl–Meyer assessment (i.e., measure of severity of motor impairment)

Before calibrating the subject-specific models, $10\times 10$-fold cross-validation accuracies of the first two runs, as well as the last two runs recorded on the first day were used to ensure that the stroke patients performed neither PM nor MI at chance level. Consequently, the study was performed using all the 6 stroke patients.

### Data processing

In this study, the FBCSP [28] algorithm was used to train the subject-specific models. First, EEG data segments from 0.5 to 2.5 s after the onset of the visual cue were used for the analysis, as a range which has been demonstrated to be effective for BCI applications [31]. Subsequent processing was carried out as described in steps 1–4, Sect. 2.1. It is noted that Chebyshev Type II was used for band-pass filtering, and for each applied CSP, *m* = 2 pairs of the spatial filters (i.e., four filters in total) were used as suggested in [28]. Finally, the LDA classifier was employed in the classification step.

## Results

### Comparing the classification results

In this subsection, the performances of the subject-specific PM and MI calibration models in detecting MI versus the rest condition were examined. Further, the proposed FB-DSA algorithm was used to reduce the dissimilarities between the MI and PM data. In the FB-DSA algorithm, to classify each new trial from the evaluation session, V in (9) was computed using the immediate past 20 trials (i.e 10 trials from each class). It should be noted that the first 20 trials of the evaluation session were only used for the adaptation, and no classification was performed on these trials. The results therefore were obtained using the reminder of the evaluation session.

#### Healthy subjects

Table 2 presents the classification accuracies of detecting MI versus the rest condition for the 16 healthy subjects, using the different calibration models. The results in Table 2 show that the calibration model using MI (MIcs) yielded, on average, higher classification accuracy (i.e., 67.44 %) compared to the calibration model using PM (PMcs) (i.e., 65.13 %) when no adaptation was applied. This result is supportive of the findings of the previous studies [24, 35], which suggest that robot-assisted PM can be used for calibrating MI-based BCI for healthy subjects. Interestingly, in some subjects the PM models considerably outperformed the MI models (e.g., *H6*). On the other hand, the results for some other exhibit a deterioration of more than 8 % in the classification accuracy when the models were calibrated using PM instead of MI (e.g., *H3*, *H8*, *H10*, *H12* and *H14*).

Table 2 shows that the proposed FB-DSA algorithm improved the classification accuracy of the PM models by an average of 4.65 %. The results also show that the PM model adapted by the FB-DSA algorithm performed better than the MI model with no adaptation by an average of 2.34 %. Furthermore, the MI models adapted by FB-DSA only slightly outperformed the PM models adapted by FB-DSA (i.e., on average $<$0.8 %).

**Table 2:** Classification accuracies of the motor imagery sessions for healthy subjects using motor imagery calibration models without and with adaptation (denoted as MIcs-No adap., and MIcs-FBDSA) and passive movement calibration models without and with adaptation (denoted as PMcs-No adap., and PMcs-FBDSA)

Subject	Healthy subjects																
	*H1*	*H2*	*H3*	*H4*	*H5*	*H6*	*H7*	*H8*	*H9*	*H10*	*H11*	*H12*	*H13*	*H14*	*H15*	*H16*	Mean
MIcs-No adap.	59.4	51.3	75.2	54.8	94.5	52.7	48.9	63.1	64.6	58.7	76.1	82.9	68.3	77.9	75.4	75	67.44
MIcs-FBDSA	61.5	66.5	86.7	63.9	89.5	54.5	57.3	64.3	60.9	63.2	76.4	76.4	75.4	77.7	80	74.5	70.55
PMcs-No adap.	63.6	54.6	51.3	57.0	92.8	62.9	51.0	54.4	58.3	50	80.3	74.2	75.8	66.2	73.7	75.8	65.13
PMcs-FBDSA	67.9	56.9	73.8	68.3	90.4	65.4	58.2	59.3	59.5	58.2	77.3	81.8	75.9	74.5	75.5	73.6	69.78

Performing a two-way repeated-measures ANOVA test with model (No-adap vs. FB-DSA) and task (MI vs. PM) as the within-subject independent variables revealed a significant main effect of Model on the classification accuracies [$F(1,15)=8.82,\,p=0.005$]. Interestingly, no significant main effect was found for Task [$F(1,15)=0.85,\,p=0.37$]. Moreover, the interaction between Model and Task was also insignificant [$F(1,15)=0.87,\,p=0.37$].

#### Stroke patients

Table 3 presents the classification accuracies of detecting MI versus the rest condition for the six stroke patients, obtained by the different calibration models. Similar to the healthy subjects, the results show that the calibration model using MI (MIcs) outperformed the calibration model using PM (PMcs) in terms of the classification accuracy. The results demonstrate that the PM model adapted by FB-DSA outperformed the MI model by an average of 4.54 %. The results also show that the MI models adapted by FB-DSA performed on average 1.5 % better than the PM models adapted by FB-DSA.

Similar to the previous section, a two-way repeated-measures ANOVA test with model (No-adap vs. FB-DSA) and task (MI vs. PM) as the within-subject independent variables was performed on the classification results obtained from the stroke patients. Interestingly, a significant main effect of Model on the classification accuracies [$F(1,15)=8.82,p=0.038$] was observed. Moreover, no significant main effect was found for Task [$F(1,15)=2.93,p=0.15$]. The interaction between Model and Task was also insignificant [$F(1,15)=0.75,p=0.43$].

**Table 3:** Classification accuracies of the motor imagery sessions for stroke patients using motor imagery calibration models without and with adaptation (denoted as MIcs-No adap., and MIcs-FBDSA) and passive movement calibration models without and with adaptation (denoted as PMcs-No adap., and PMcs-FBDSA)

Patient’s code	Stroke patients						
	A006	A018	A019	A024	A028	A031	Mean
MIcs-No adap.	81.9	65	64.4	57.5	85.6	90.6	74.16
MIcs-FBDSA	85.7	69.3	86.4	67.1	85.7	87.1	80.21
PMcs-No adap.	85.6	55	51.3	55.6	61.3	93.8	67.10
PMcs-FBDSA	84.3	67.9	80	59.3	87.86	92.86	78.70

We also performed a 3-way mixed design ANOVA with model (No-adap vs. FB-DSA) and task (MI vs. PM) as the within-subject independent variables and group (healthy vs. stroke subjects) as the between subject independent variable. The results showed a significant main effect of Model on the classification results [$F(1,20)=16.88,p=0.001$]. Interestingly, the interaction between Model and Group tended to be significant [$F(1,20)=3.75,p=0.06$]. This indicates a significant larger increase in the accuracy of the stroke group compared to the healthy group when applying adaptation. As a result, the big drop in the performance of PM model compared to the MI model in the stroke patients was compensated after applying the proposed adaptation algorithm. On the contrary, the main effect of Task [$F(1,20)=3.28,p=0.09$], the interaction between Group and Task [$F(1,20)=0.67,p=0.42$], and the interaction between Task and Model [$F(1,20)=1.45,p=0.24$] were not significant.

#### Comparing with two existing non-adaptive algorithms:

In this part, the performance of the proposed FBDSA algorithm is compared with two existing non-adaptive algorithms that are computationally as efficient as the proposed algorithm. In the first approach, the FBCSP algorithm was trained using only the first 20 trials of the test session that were previously used only for adaptation. This calibration model is called FBCSP-20. In the second approach, again the first 20 trials of the test session were used for training a FBCSP model, whereas the covariance matrices for computing CSP were estimated using the BC shrinkage algorithm [36]. This calibration model is called FBCSP-20Shrink. BC shrinkage is a computationally efficient algorithm to estimate covariance matrices without requiring time-consuming cross-validation procedures. Interestingly, BC shrinkage is shown to be very effective when the number of samples is limited [36]. To apply the BC shrinkage algorithm, as suggested in [36], for each subject, the average of the class covariances of the other subjects was used as the shrinkage target.

**Fig. 4:**
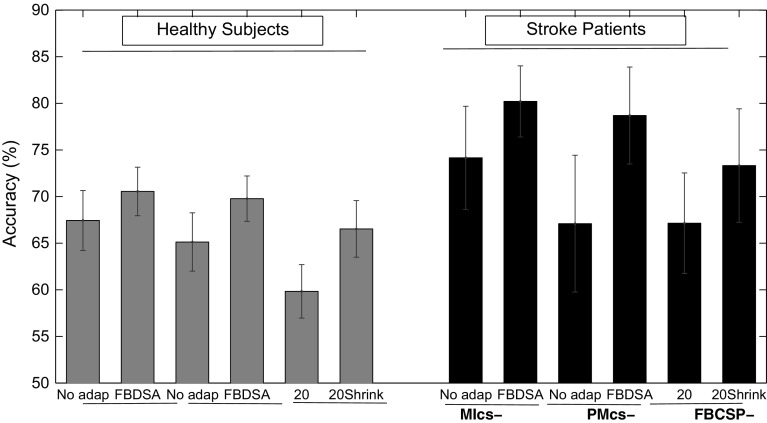
Comparison between the performance of the proposed FBDSA algorithms using MI and PM calibration models with FBCSP algorithm trained on 20 first trials of the test session with and without the BC shrinkage regularization algorithm

Figure 4 shows that the proposed FBDSA algorithm trained using either the MI or PM data outperformed the FBCSP-20 and FBCSP-20Shrink algorithms. The paired t-tests on the healthy group showed that FBCSP-20 performed significantly worse than MIcs-No adap [$t(15)=-3.85, p=0.002$], MIcs-FBDSA [$t(15)=-4.82, p<0.001$], PMcs-No adap [$t(15)=-6.26, p=0.007$], and PMcs-FBDSA [$t(15)=-3.14, p<0.001$]. Furthermore, MIcs-FBDSA [$t(15)=-2.63, p=0.019$] and PMcs-FBDSA [$t(15)=-2.27, p=0.038$] significantly performed better than FBCSP-20Shrink. In the stroke group, the paired t-test showed that FBCSP-20 performed significantly worse than MIcs-FBDSA [$t(5)=-4.3, p=0.008$], and PMcs-FBDSA [$t(5)=-3.29, p=0.002$]. Moreover, a significant difference between FBCSP-20Shrinkage and PMcs-FBDSA was observed [$t(5)=-2.59, p=0.04$[, whereas the difference between FBCSP-20Shrinkage and MIcs-FBDSA tended to be significant [$t(5)=-2.39, p=0.06$].

#### Discussion on the classification results

Our results show a potential mismatch between the collected PM data in the calibration session and the collected MI data in the evaluation session. This mismatch may have arisen due to differences in the EEG patterns produced during PM and those produced during MI. Other intersession variations may also be responsible (e.g., task involvement, attention, placement or impedance of the electrodes etc), leading to deterioration in the BCI performance.

Our results show that for the healthy subjects the MI models on average performed 2.31 % better than the PM models (see Table 2), whereas this difference increased to 7.06 % in the stroke patients. Indeed, for the stroke patients although the PM model performed at a level better than chance, the average 7.06 % drop in the performance compared to the MI model suggests that PM as utilized could not be an acceptable substitute for an MI calibration step. However, when the proposed FB-DSA algorithm is applied to the PM model, the results indicate a compelling compensatory effect.

Our patients in our informal talks confirmed that performing passive movement was less mentally fatiguing for them. Thus, when no previous data are available for a studied patient, we suggest using PM data for calibration and the proposed FB-DSA algorithm for adaptation, resulting in a less tired patient for the actual BCI therapeutic interaction. Furthermore, since PM exercises are a part of normal stroke rehabilitation [25], PM data can be collected in a previous physical therapy session. Otherwise, if there are some previously collected MI data belonging to the same patient, our results suggest using the available MI data for calibration along with the proposed FB-DSA for reducing inter-session variations.

It is noted that our conclusions are limited to the data collected from only 6 stroke subjects. Thus, there is a need to further investigate our findings with larger cohorts.

### Disparity between PM and MI data in stroke and healthy subjects

The results in 4.1 motivated us to investigate whether the disparity between the PM and MI data is stronger in the stroke subjects compared to the healthy subjects.

#### Event-related spectral perturbation

In the first investigation, the grand mean event-related spectral perturbation (ERSP) [37], time locked to the cue time, was used to compare the PM and MI tasks for the healthy and stroke subjects. ERSP is a 2-D (frequency-by latency) image of average changes in the spectral power (in dB) from a baseline. Calculating an ERSP typically requires computing the power spectrum over a sliding latency window, then correcting baseline by subtracting the pre-stimulus power spectrum, and finally averaging across all the data trials.

**Fig. 5:**
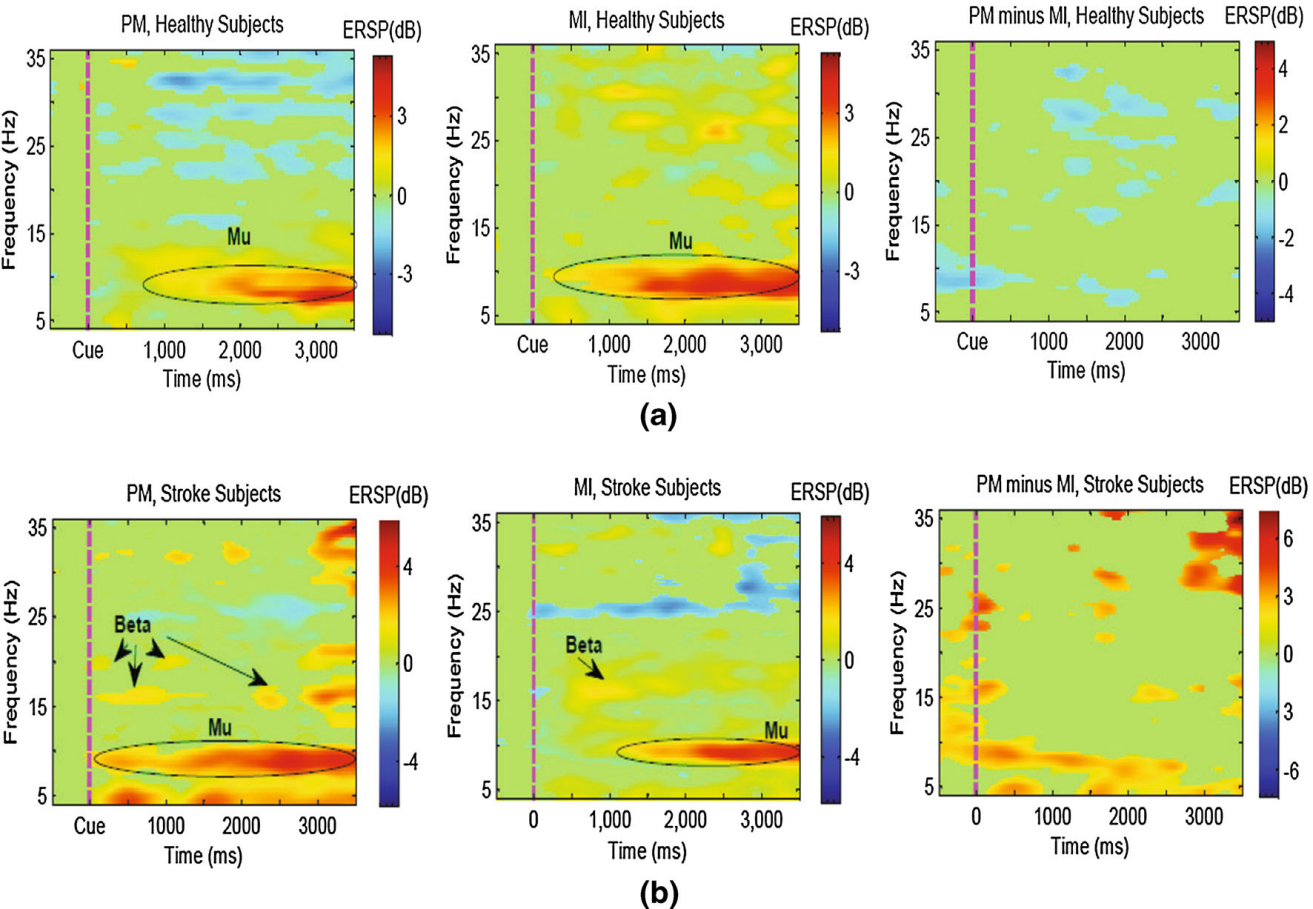
The event-related spectral perturbation (ERSP) images for the passive movement (PM), motor imagery (MI) and their differences, for **a** the 16 healthy and **b** six stroke subjects. The ERSP images were plotted at the corresponding activated motor regions (ie. channels C3 and C4 for *right* and *left* hand tasks, respectively). The *dashed lines* denote the cue time. In MI and PM ERSP images, the *non-green* pixels indicate the areas that the power spectrum is significantly different from the pre-cue baseline. In PM minus MI ERSP images, the*non-green* pixels indicate the areas that the power spectrum between PM and MI is significantly different ($p < 0.01$). **a** Healthy subjects. **b** Stroke patients

Figure 5 presents the ERSP images obtained by grand averaging the data recorded from channel C3 for the right hand tasks and the data recorded from channel C4 for the left hand tasks. The ERSP images were plotted using the *newtimef* function in EEGLAB toolbox [38]. In the MI and PM ERSP images, red indicates enhancement of activity (increase of power) with respect to the pre-cue baseline (i.e., starting from 200 ms before the cue), and blue indicates suppression of activity with respect to the pre-cue baseline. All the non-green pixels of the MI and PM ERSP images show significant (two-tailed permutation test, $p < 0.01$) post-stimulus increases or decreases (see color scale) in the spectral power compared to the averaged 200-ms pre-stimulus spectral power. In the PM minus MI ERSP images, red indicates higher activity (power) in PM compared to MI, and blue indicates higher activity in MI compared to PM. All the non-green pixels of the PM minus MI ERSP images show the areas that the spectral powers are significantly different between MI and PM (two-tailed permutation test, $p < 0.01$).

Figure 5a shows that both PM and MI tasks significantly increased the mu power (i.e 8–13 Hz) in the healthy subjects. However, the increase in power in MI is slightly greater compared to PM. This could be due to the very simple passive movement used in this study, whereas the imagined motor movements over the MI task were most-likely more complicated. In the same line, Fig. 5b shows that the PM and MI tasks yielded significant increase in the mu and beta (i.e., 13–30 Hz) powers. In the stroke patients, PM yielded considerably stronger enhancements in the mu and beta powers compared to the MI task. Interestingly, PM also yielded an enhancement in the theta power (i.e., 4–8 Hz).

The last ERSP image in Fig. 5a shows that the PM and MI signals obtained from the healthy subjects were significantly different in some time/frequency points, particularly 1 s after the cue. The last ERSP image in Fig. 5b reveals that the differences between PM and MI were much stronger in the stroke patients compared to the healthy subjects (see the scale). Importantly, the mu power was significantly different between the PM and MI data in the stroke patients.

#### KL divergence between PM and MI data

In the second investigation, the KL divergence between the PM and MI data filtered by the most discriminative frequency band (i.e., subject-specific) was calculated for each subject as given in (6). The reason behind this investigation is that in the applied FBCSP algorithm the features obtained by the most discriminative frequency band are used for classification. As mentioned in Sect. 2.1, the most discriminative frequency band is the one generating the feature with the highest mutual information. Indeed, unlike the ERSP images, this method measures the disparity between the PM and MI data using all (or a group) of channels.

**Fig. 6:**
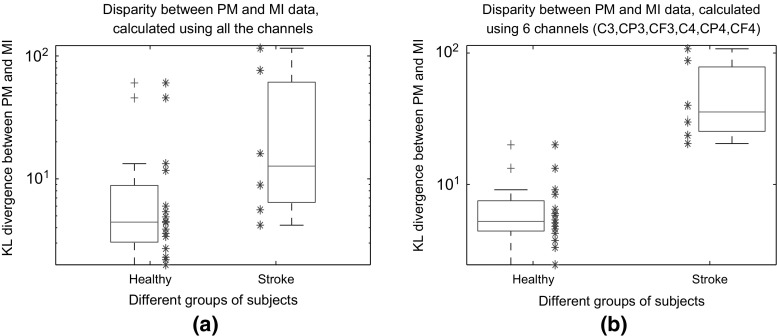
Comparing the disparity between PM and MI data in the stroke and healthy subjects obtained by the KL divergence. To calculate the KL divergence **a** used all the channels, and **b** used six channels in the motor cortex area, namely C3, CP3, CF3, C4, CP4, CF4. Each star denotes a subject. The* boxplots* of the obtained results were plotted to ease the comparison

Figure 6 compares the KL divergence between the PM and MI data in the healthy and the stroke subjects. Each star corresponds to a subject. The boxplots of the obtained results were also depicted to ease the comparison between the healthy and stroke subjects. In Fig. 6a the KL divergence was obtained using all the 27 channels, while in Fig. 6b only 6 channels in the motor cortex area were used to obtain the KL divergence (i.e C3, CP3, CF3, C4, CP4, CP3). The *Y*-axes of Fig. 6 have been drawn in the log scale to be more presentative.

Using all the channels, Fig. 6a shows that the difference between the PM and MI data is on average larger in the stroke subjects compared to the healthy subjects. However, according to the independent samples *t* test, the difference between the results of these two groups was not statistically significant [$t(20)=1.86, p=0.07$], since there were a few healthy subjects with high KL divergences between their PM and MI data. Indeed, the obtained KL values measured not only the inherent dissimilarities between the PM and MI data but also other inter-session variations (e.g task involvement, electrode impedance) that may not be negligible. Supporting this fact, our investigation showed that in one of the healthy subjects an EEG channel became loose at the middle of the PM session, and consequently caused a large KL divergence between the PM and MI data. Thus, to reduce the effect of other inter-session non-stationarities, in Fig. 6b we focused on the motor cortex area, and obtained the KL divergence using only six channels (i.e., C3, CP3, CF3, C4, CP4, CP3). The results in Fig. 6b showed that in the stroke subjects the difference between the PM and MI data recorded over the motor cortex area was statistically stronger compared to the healthy subjects (i.e independent samples *t* test, $t(20)=2.14,p=0.04$).

#### Discussion on the disparity between PM and MI

According to the study presented by Galán et al. [39], the lack of sensory feedback in the MI data might cause some fundamental differences between MI and PM. To further investigate this issue, the ERSP images for centro-parietal electrodes which are closer to the sensorimotor cortex (i.e., CP3 for the right hand tasks and CP4 for the left hand tasks) were plotted. The ERSP images were relatively similar to the ones in Fig. 5 confirming the available differences between the PM and the MI data over the sensorimotor cortex. Due to the limited space these ERSP images are not included in the paper.

According to Fig. 5, it appears that most of the interesting different activities between the PM and MI data is in the mu band. In order to find which frequencies are more different between MI and PI, we looked at the data space adaptation matrices (${\mathbf {V}}^{*}_{\rm b}$) calculated for the different frequency bands. If the MI and PM for a specific frequency band are similar, the ${\mathbf {V}}^{*}_{\rm b}$ matrix is close to the identity matrix. To measure the differences between PM and MI in each frequency band, the Frobenious norm between ${\mathbf {V}}^{*}_{\rm b}$ and the identity matrix was calculated as $\Vert {\mathbf {V}}^{*}_{\rm b}-\mathbf {I}\Vert _{F}$, where **I** is the identity matrix and $\Vert .\Vert _{F}$ denotes the Frobenious norm.

Interestingly, the results showed that for all the stroke patients the mu rhythm had the largest difference between the PM and MI data. Besides, the theta rhythm was observed as the second largest different frequency band in 4 out of 6 patients. Several studies reported the theta enhancement during working memory, memory decoding and retrieval process. A recent study also reported the theta enhancement during the initiation of movement, and linked it to spatial exploration and self-direction learning [40]. This initial interesting findings should be further explored using a large number of stroke patients in future.

In the healthy subjects, mu, alpha, and the lower beta (i.e., 12–16 Hz) showed the largest difference between PM and MI for 8, 4 and 2 subjects, respectively. For the remaining two subjects the 28–32 Hz band and the 36–40 Hz band presented the largest difference. Thus, our results suggest that in most of the subjects the main discriminative frequency bands between PM and MI were mu and theta, respectively. However, since the proposed FB-DSA adaptation is a very computationally fast algorithm, it might not be worth to just apply the adaptation algorithm on one or two bands.

In summary, our results in Sect. 4.2 suggest that due to a stronger difference observed between the PM and the MI data in the stroke patients, there might be an increased need to use adaptive algorithms such as the proposed FB-DSA algorithm in BCI-based stroke rehabilitation calibrated from PM data.

### Impact of FB-DSA on the feature space

To better understand the impact of the proposed FB-DSA algorithm on the classification accuracies of MI-BCIs calibrated using passive data, the training features and the evaluation features before and after applying the FB-DSA algorithm were plotted for the patient A019. Adapting the PM model of this patient by the proposed FB-DSA algorithm resulted in the highest improvement in the classification accuracy, which was 28.75 % (see Table 3).

**Fig. 7:**
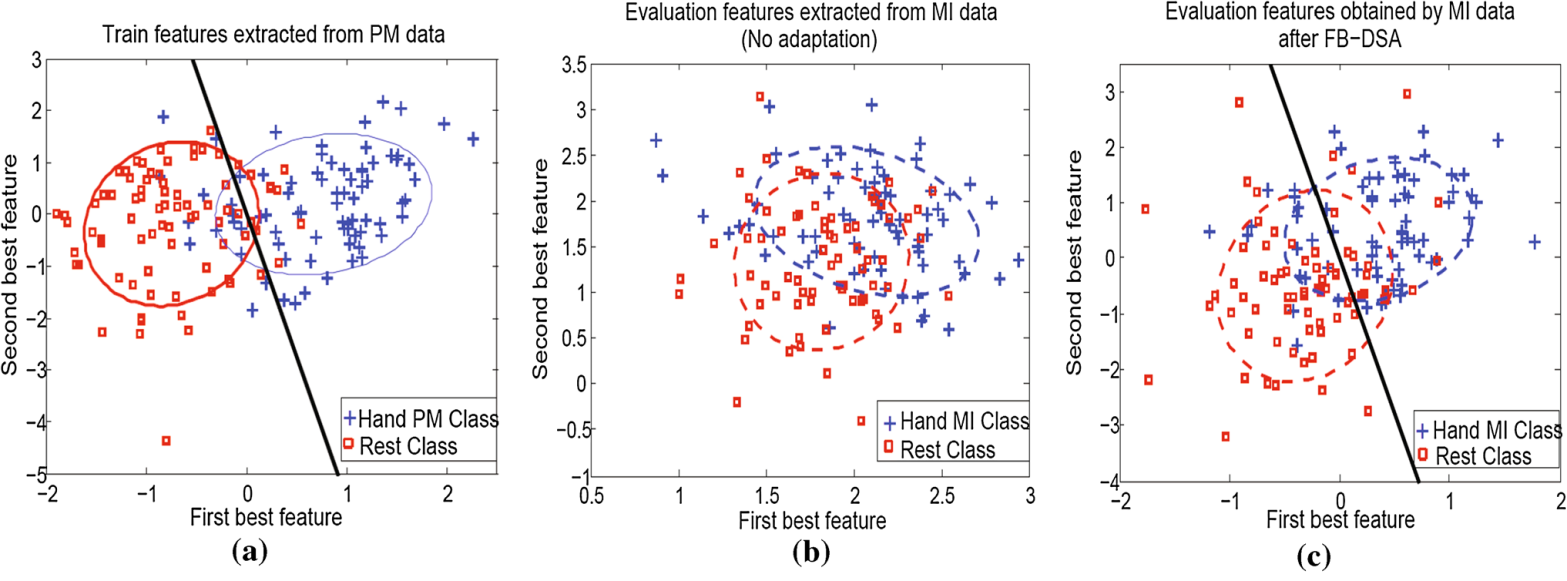
Distributions of the two best features obtained by the PM calibration model, for: Patient A019. **a** The train features extracted from the PM data, subsequently **b** and **c** The evaluation features extracted from the MI data before and after adapting by the proposed FB-DSA algorithm. The* blue crosses* and the* red squares* denote the features of the hand MI/PM and the rest class respectively. The *black line* represents the LDA hyperplane obtained by the train data

Figure 7a shows the train features of the PM model extracted from the PM data. Figure 7b, c, respectively show the evaluation features of the PM model extracted from the MI data before and after applying the FB-DSA algorithm. For ease of visualization only two features which had the highest mutual information on the train data were plotted. Moreover, the features were plotted after the normalization process. The blue crosses and the red squares denote the features of the hand MI/PM and the rest class respectively. The black line represents the LDA hyperplane obtained using the training data. Figure 7 shows that there were big changes between the distributions of the training features and the evaluation features before applying FB-DSA, that resulted in the inferior classification accuracy. In contrast, the differences between the train and the evaluation features after the proposed FB-DSA algorithm were considerably reduced. The FB-DSA algorithm not only compensated the shift in the feature spaces but also increased the discrimination between the two classes of the evaluation features. Thus, the classification accuracy was substantially improved.

### Number of trials for adapting the PM model by FB-DSA

**Fig. 8:**
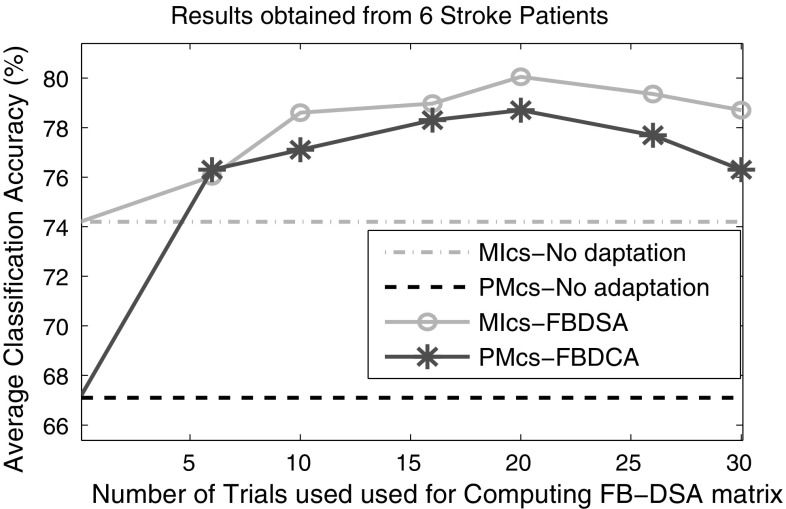
Influence of the number of trials used for computing FB-DSA matrices on the classification results of the stroke patients. MIcs and PMcs respectively denote motor imagery and passive movement calibration models applied on the remaining 210 trials of the evaluation sessions

In this subsection, we examined the influence of the number of trials used for computing the FB-DSA transformation matrices on the classification results of the PM and MI models in BCI-based stroke rehabilitation. For this purpose, the data from the 6 stroke patients were only used. Figure 8 shows the average classification accuracy of the PM and MI models adapted by the proposed FB-DSA algorithms for the six stroke subjects as a function of the number of trials used for computing ${\mathbf {V}}^{*}_{\rm b}$ in (9). Thus, to classify each new trial in the evaluation session, ${\mathbf {V}}^{*}_{\rm b}$ was computed using a number of immediate past trials varying from 0 to 30. It is noted that in this experiment the first 30 trials of the evaluation sessions (i.e., 15 trials from each class) were only used for computing the FB-DSA transformation matrices, and the classification was performed on the remaining 210 trials of the evaluation sessions.

Figure 8 shows that increasing the number of trials used in the FB-DSA algorithm up to around 20 (i.e., 10 trials from each class) improved the average classification accuracy of both the PM and MI models. This improvement would be due to better estimations of the covariance matrices in (9) using more trials. In contrast, a further increase in the number of trials to 30 caused a decrease in the average accuracy. This could be due to the fact that increasing the number of trials reduces the influence of the recent trials in computing ${\mathbf {V}}^{*}_{\rm b}$. Figure 8 also shows that when six trials or more were used to compute the FB-DSA transformation matrices, the PM model adapted by FB-DSA averagely outperformed the MI model with no adaptation.

In a BCI-based stroke rehabilitation, it would be desirable if we could provide appropriate feedback to the patient from the very beginning. Overall, our results suggest that the BCI-based stroke rehabilitation session with appropriate feedback can be started after collecting just a few trials (e.g., only 3 trials from each class). This is achieved by a subject-specific model calibrated from PM data that is continually adapted using the proposed FB-DSA algorithm. As such, before collecting 20 evaluation trials (i.e., 10 trials from each class), the PM model classifies an upcoming trial using the FB-DSA transformation matrices computed from all the previous trials. After reaching 20 trials, to classify each new trial, the FB-DSA transformation matrices are computed using the immediate past 20 trials.

## Conclusion

This paper investigated the effectiveness of calibrating EEG-based motor imagery BCIs using passive movement. For this purpose, a new algorithm called FB-DSA was proposed to linearly transform the filter bank band-passed MI data, such that the distribution difference between the MI and PM data is minimized. The proposed algorithm was evaluated using data from six stroke patients and 16 healthy subjects. The design of this study for both the stroke and healthy subjects was based on the use of motor imagery-based BCI for stroke rehabilitation [5]. The EEG data were collected during MI or PM of the chosen hand versus the rest condition for the healthy subjects, and the stroke affected hand versus the rest condition for the patients.

Our results suggest using PM data for calibration and the proposed FB-DSA algorithm for adaptation when no previous data are available for a studied patient. Importantly, collecting PM data for calibration leads to a less mentally tired patient for the actual BCI therapeutic interaction. Furthermore, since PM exercises are a part of normal stroke rehabilitation, PM data can be collected in a previous physical therapy session. On the other hand, if there are some previously collected MI data belonging to the same patient, our results suggest using the available MI data for calibration along with the proposed FB-DSA for reducing inter-session variations. We also provided some analytical evidence suggesting that the disparity between the MI and PM data could be significantly stronger in the stroke patients compared to the healthy subjects. Thus, there might be an increased need to use adaptation algorithms such as the proposed FB-DSA algorithm in BCI-based stroke rehabilitation calibrated from PM.

Overall, the results showed that a BCI-based stroke rehabilitation session with appropriate feedback could be reliably started after collecting just a few trials (e.g., only 3 trials from each class). This could be achieved by using a subject-specific model calibrated from robot-assisted PM data that was continually adapted using the proposed FB-DSA algorithm.
